# The Use of Negative Pressure with Instillation and Dwell for the Treatment of Necrotizing Fasciitis

**DOI:** 10.7759/cureus.3515

**Published:** 2018-10-29

**Authors:** Kersten Reider, Elizabeth McElroy, Stormy Lemay

**Affiliations:** 1 Wound, Ostomy, Continence Care / Nursing Administration, Reading Health System, Reading, USA

**Keywords:** negative pressure wound therapy, instillation, nonviable tissue, necrotizing fasciitis, wound cleansing

## Abstract

The human body is a complex, multisystem organism that can manifest disease processes in a multitude of ways. Over the decades, technological advancements have allowed us to make precise diagnoses so that clinicians can thoroughly treat the underlying cause. Frequently these disease processes require surgical intervention to eliminate the progression and provide the patient with positive outcomes. When surgical intervention is required, the patient is often left with large complex wounds. Just like medical advancements, wound care modalities have made vast technological improvements. Wounds previously being treated with simple but labor-intensive treatments such as gauze packings and return operating room interventions, can now be treated with negative pressure wound therapy combined with instillation and dwell (NPWTi-d). This therapy combines the benefits of negative pressure while cleansing the wound through the instillation of a topical wound cleanser in a controlled environment.

In this case review, we will highlight a case of necrotizing fasciitis in which surgical intervention was required and negative pressure wound therapy with instillation and the use of reticulated open cell foam dressing with through holes (ROCF-CC) was utilized. Negative pressure with instillation was used to remove infectious material and other nonviable tissue from the wound base while promoting granulation tissue production. By utilizing this treatment, we were able to decrease the patient's return trips to the operating room (OR), enhance granulation tissue production, and ultimately achieve positive patient outcomes.

## Introduction

Necrotizing fasciitis is a rare but life-threatening infection of the skin, soft tissues, and muscles, which tends to progress rapidly through the fascia planes causing gradual destruction of the fascia. Most cases present with anaerobic bacteria that proliferate in a hypoxic environment and produce gas that accumulates in the soft tissue spaces, giving a characteristic image of gas gangrene on plain X-rays [1]. Early diagnosis of necrotizing fasciitis is imperative. Extensive wide debridement of all devitalized tissue, usage of broad-spectrum antibiotics, and management of underlying disease are the main cornerstones for management of the disease [2]. Many times, these patients cannot undergo serial debridements due to hemodynamic instability and overall poor condition. Several studies in the general surgery, orthopedic, and gynecological literature support the use of negative pressure wound therapy (NPWT) device for fast and effective wound closure [1]. Negative pressure treatment can remove exudate, reduce bacterial contamination, and preserve vital tissues in the wound. Just like advances in medical technology, NPWT has made vast technological advancements with the introduction of negative pressure with instillation and dwell (NPWTi-d). This therapy has proven to decrease return trips to the operating room (OR) for debridement, increase healing rates, and decrease length of hospitalization [3]. Then in 2017, a new reticulated open-cell foam dressing with through holes (ROCF-CC) was introduced. NPWTi-d using ROCF-CC is used to assist with wound cleansing by removing thick wound exudate and infectious materials [4]. As mentioned previously, many times patients cannot undergo further debridement and are left with non-viable tissue in the wound. NPWTi-d with ROCF-CC can be applied to facilitate the loosening, solubilizing and detachment of viscous exudate, dry fibrin, wet slough, and other infectious material prior to OR debridement, after OR debridement, or in cases when surgical debridement is not an option [5].

## Case presentation

**Figure 1:**
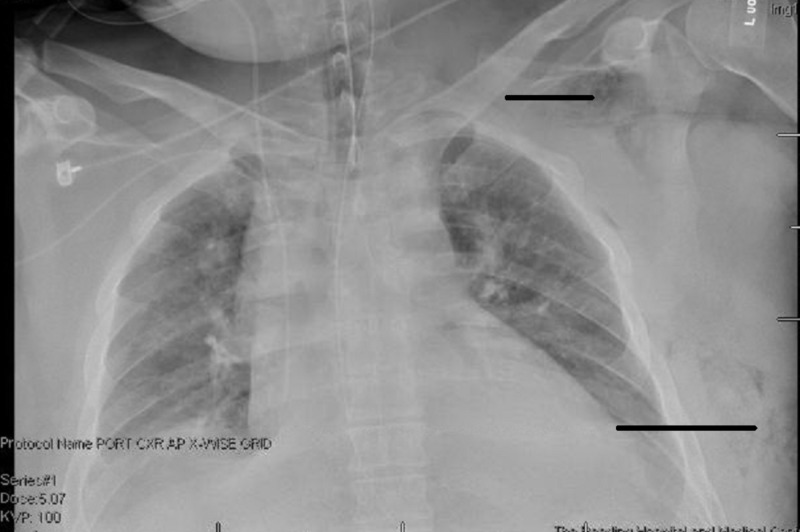
X-ray that shows soft tissue gas in the left chest

A 36-year-old Caucasian male presented to the emergency room complaining of a one-day history of abdominal pain. His main symptoms were that of nausea and vomiting, but he also reported periods of diarrhea. In the emergency room, his initial evaluation was significant for lab studies demonstrating a mild metabolic acidosis with a bicarb of 16.6 mEq/L, an elevation of his creatinine to 1.93 mg/dL, and a serum lactate level of 5.4 mmol/L. A computed tomography (CT) scan of the abdomen and pelvis was obtained and reviewed. The appendix was thought to be normal. There was no evidence of free intraperitoneal air, abscess, or volvulus. There did appear to be evidence of enterocolitis involving the ilium, cecum, and the proximal ascending colon. There was no evidence of pneumatosis or obstruction. Over the course of 12 hours the patient underwent conservative medical management, which included intravenous hydration, intravenous steroids, Toradol and Dilaudid for pain management, and the occasional dose of Ativan for agitation. Failure of conservative management along with medical decline lead to an exploratory laparotomy with a right hemicolectomy for an ileocolic intestinal infarction. Postoperative repeat CT scan of the abdomen incidentally showed pulmonary nodules in the lower lobe. A CT scan of the chest was then obtained, which revealed air in the left chest wall and axilla (Figure 1). On physical exam there was very subtle mottling of the left shoulder. The patient was emergently taken back to the OR for further exploration of the shoulder with excisional debridement. Seropurulent fluid and extensive subcutaneous emphysema along the fascial planes of the left chest wall were consistent with necrotizing fasciitis. Both wounds were packed with a gauze bandage roll soaked in saline and then the patient was transferred back to the intensive care unit.

Over the course of 15 days the patient underwent repeat trips to the OR for re-exploration, incision and drainage of the wounds, and dressing changes (Table 1). On postoperative day 17, the wound, ostomy, continence nurse (WOCN) was consulted to evaluate the wound and make treatment recommendations.

**Table 1:** Treatment progression OR=Operating room, NPWT=Negative pressure wound therapy, NPWTi-d=Negative pressure wound therapy with instillation and dwell.

Treatment day	Procedure	Dressing
Initial OR visit	Excisional Debridement	Gauze bandage roll soaked in saline
Day 2	Incision and Drainage	Gauze bandage roll soaked in saline
Day 4	Incision and Drainage	NPWT placement
Day 8	Incision and Drainage	NPWT placement
Day 10	Bedside dressing change	NPWT placement
Day 12	Dressing change in the OR	NPWTi-d applied
Day 14	Dressing change in the OR	Gauze bandage roll soaked in Dakin’s solution
Day 15	Bedside dressing change	Collagenase ointment with saline soaked gauze bandage roll

**Figure 2:**
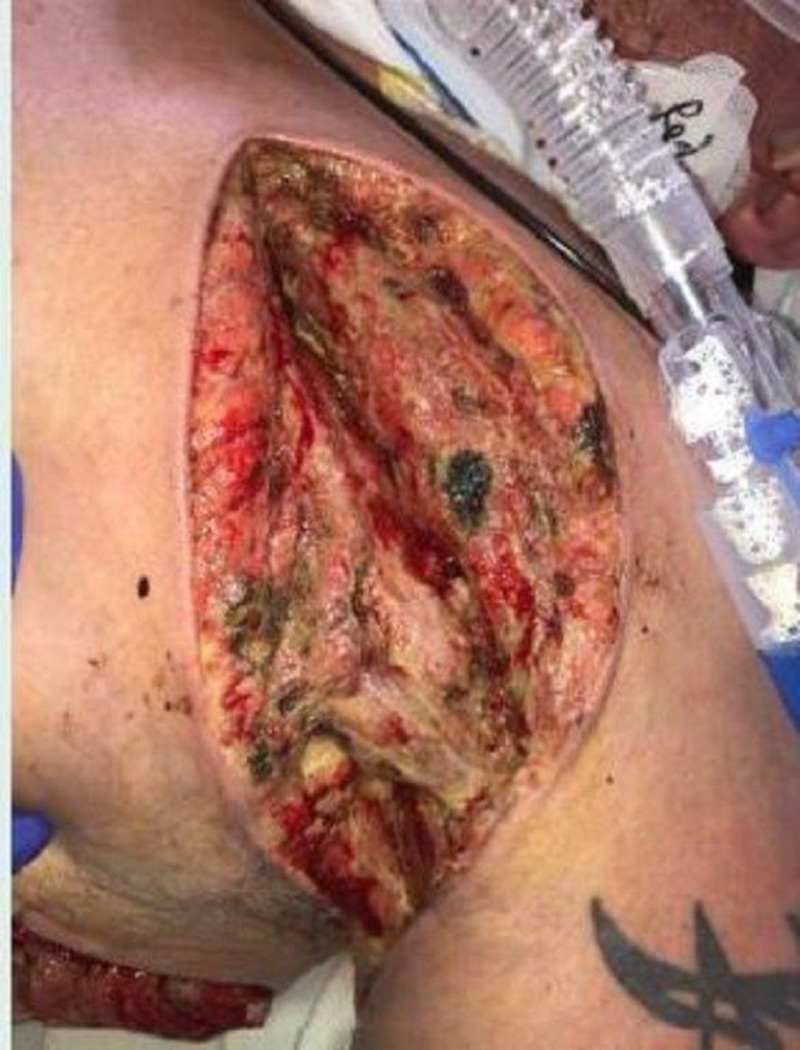
Left axilla wound

**Figure 3:**
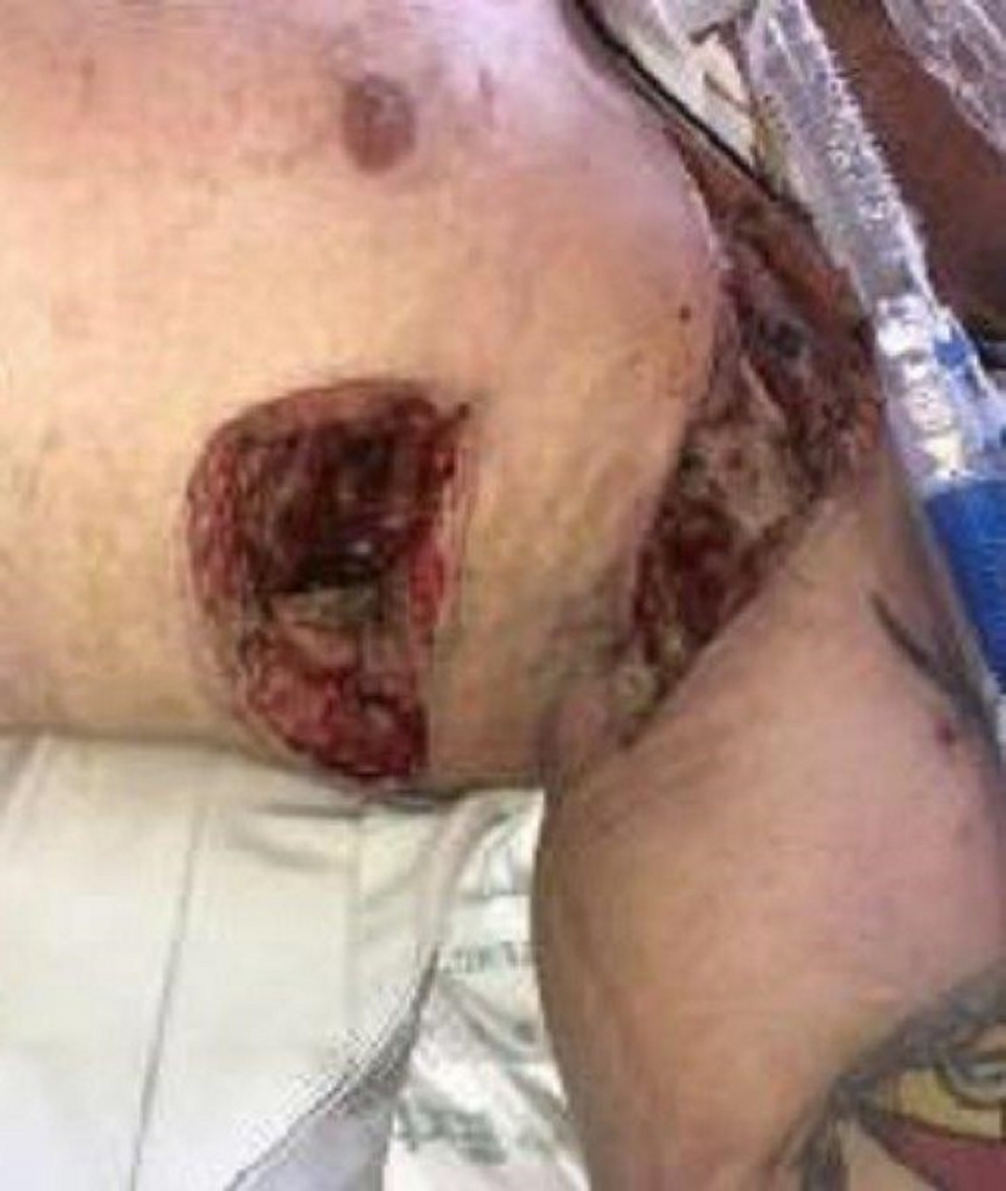
Both axilla and chest wounds

**Figure 4:**
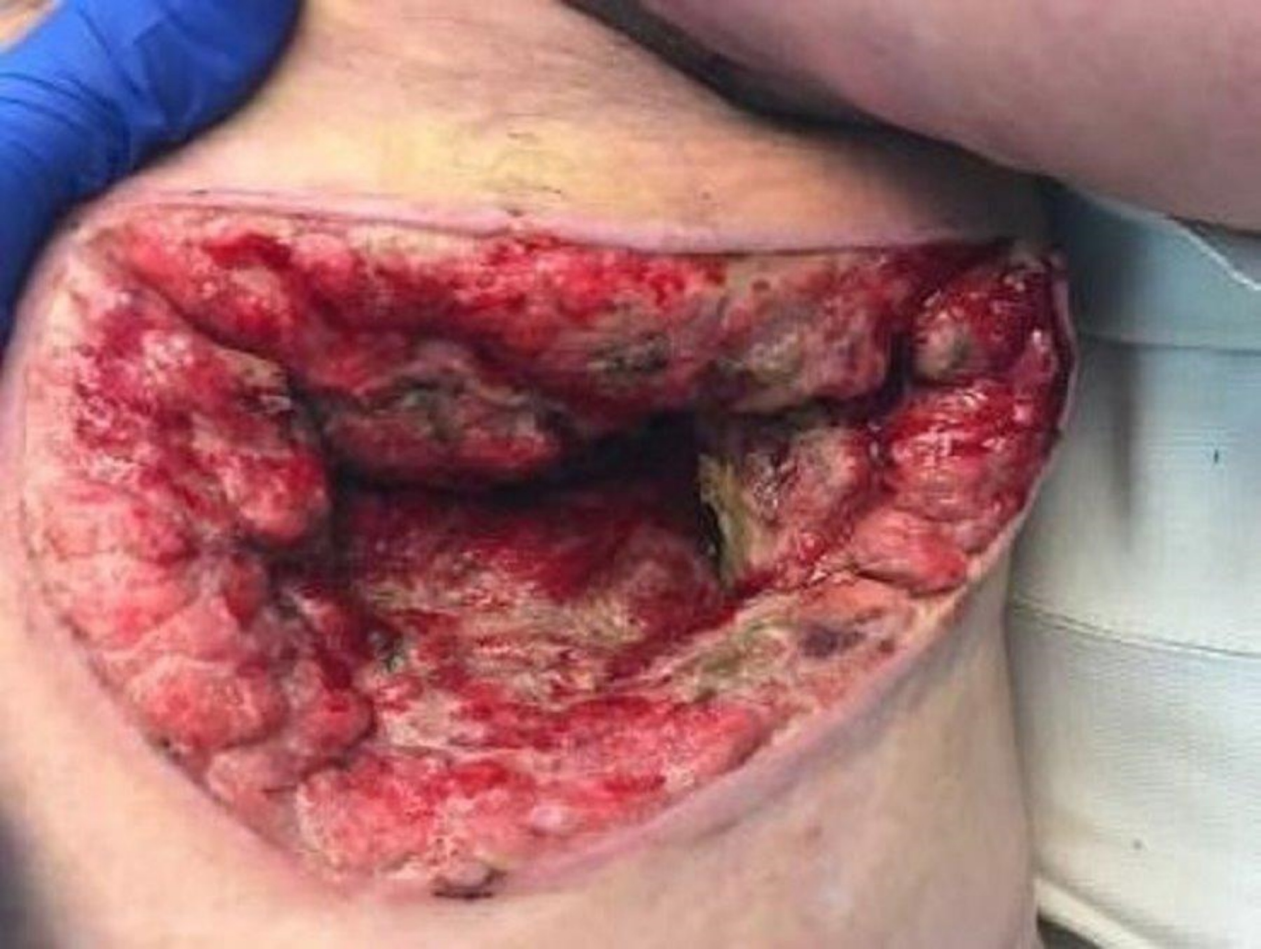
Left chest wound

On exam, there was still a significant amount of nonviable tissue in the base of the left chest and axilla wound. The left chest wound measured 8 x 19 x 8 cm and the left axilla wound measured 6.5 x 25 x 4 cm. NPWTi-d with ROCF-CC was applied to these wounds to aid in the removal of the nonviable tissue. Figures 2-4 show the presentation of the wounds prior to the application of the NPWTi-d with ROCF-CC.

**Figure 5:**
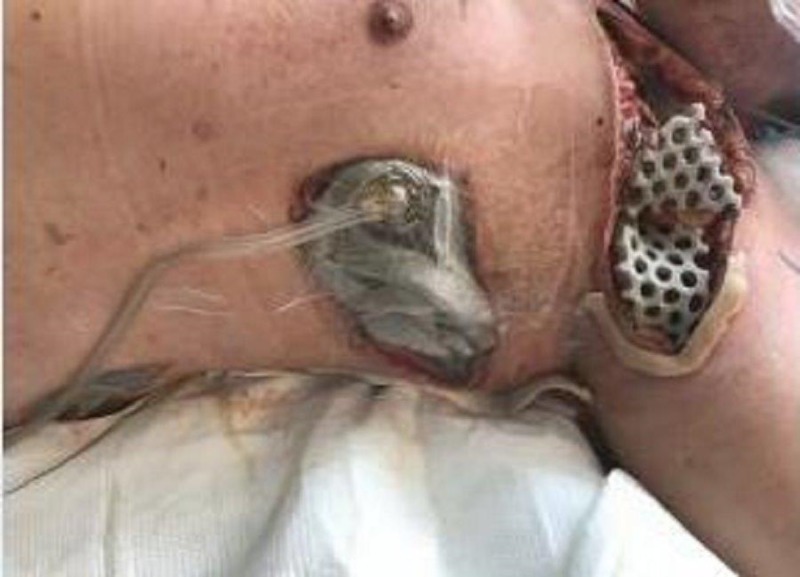
Application of NPWTi-d with ROCF-CC

During the application of the NPWTi-d with ROCF-CC, care was taken to ensure that the contact layer of the dressing was in contact with the entire wound base. Barrier rings were applied to the skin creases along with any area that presented as a high risk for inadequate seal of the drape (Figure 5). The therapy settings for the left axilla were 85 ml of hypochlorous acid solution (Vashe®, SteadMed, Fort Worth, TX), dwell time of 10 minutes with NPWT every hour at -125 mmHg. Therapy settings for the left chest wound were 50 ml of hypochlorous acid solution, dwell time of 10 minutes with NPWT every hour at -125 mmHg.

**Figure 6:**
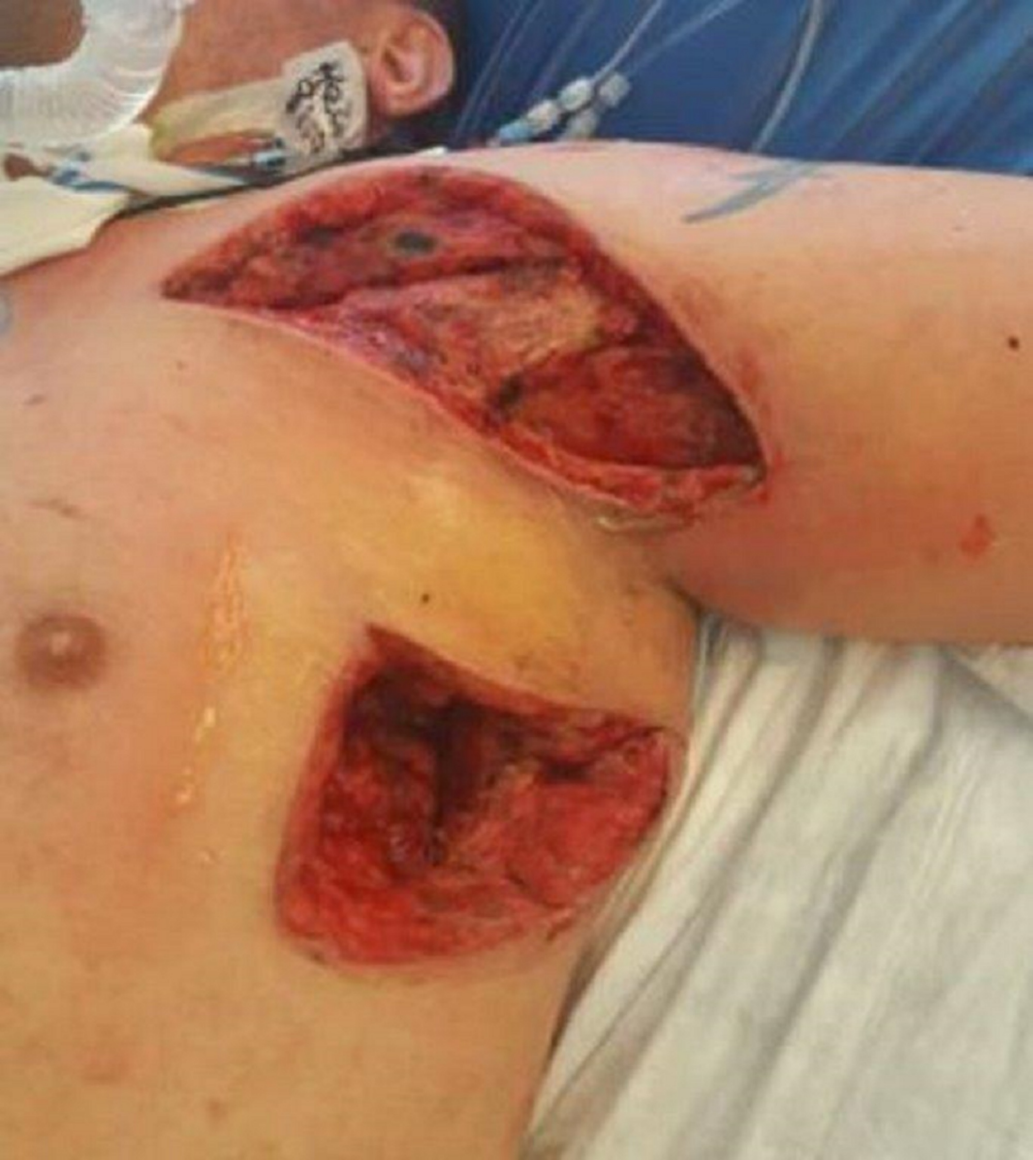
First dressing change after application of NPWTi-d with ROCF-CC

During the first dressing change after the application of the NPWTi-d with ROCF-CC, there were notable improvements in the quality of the tissue in the wound bed (Figure 6). The left chest wound bed was noted to have 100% red healthy tissue and the left axilla wound bed had a decrease in nonviable tissue. There was, however, seropurulent drainage noted deep within the intramuscular structures of the left axilla wound.

**Figure 7:**
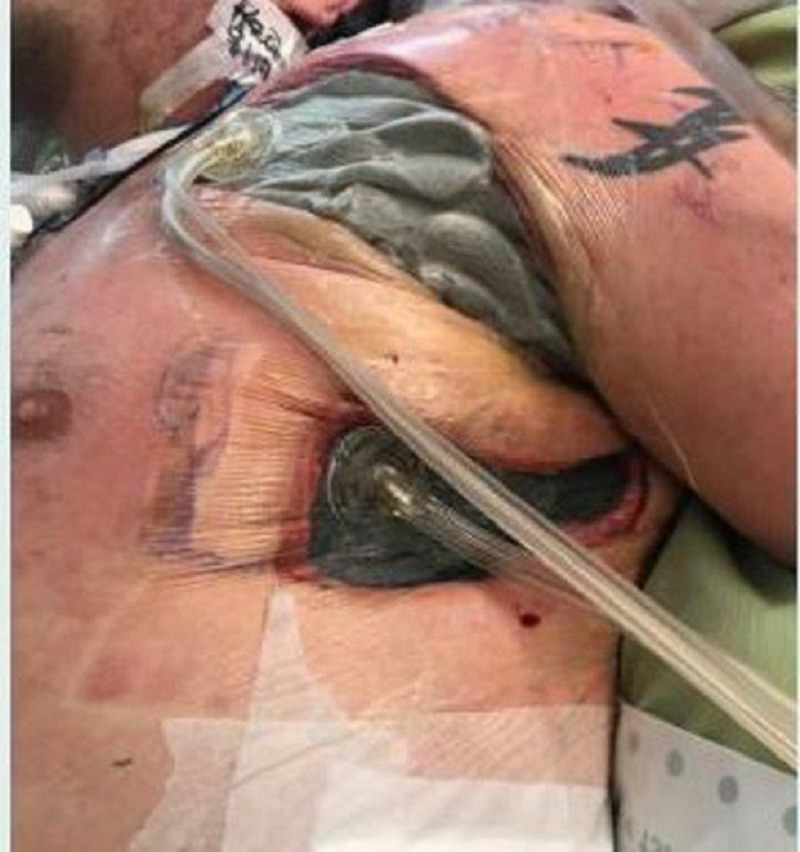
NPWTi-d with ROCF-CC to axilla wound, NPWTi-d to chest wound

NPWTi-d with ROCF-CC was reapplied to the axilla wound ensuring that the contact layer was placed deep into the wound bed to facilitate the removal of the seropurulent drainage. The left chest wound was transitioned to NPWTi-d to continue to promote granulation tissue (Figure 7). The instillation solution was switched to normal saline due to blockage alarms we encountered with the hypochlorous acid solution without resolution.

**Figure 8:**
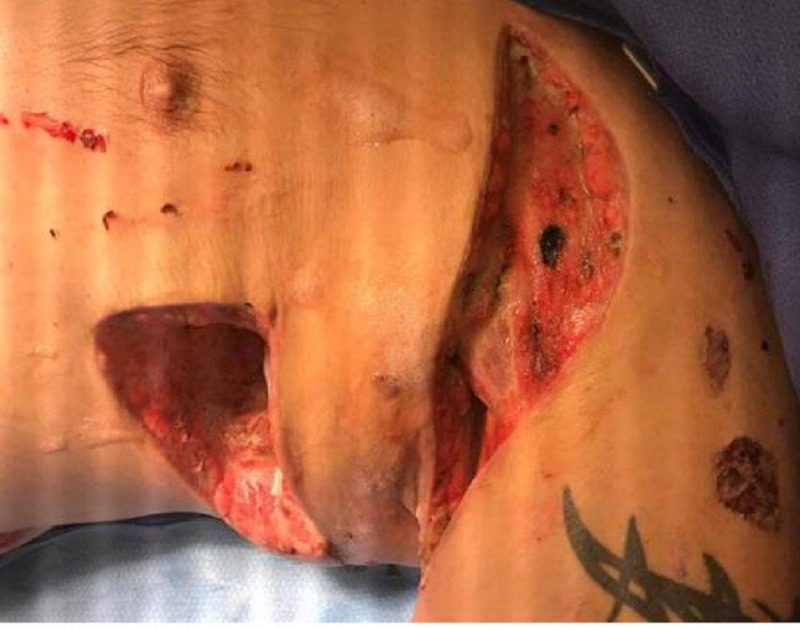
Granular tissue noted in both wound bases

The current treatment regimen was continued for two days until the next dressing change. At that time both wound bases were 100% red with granulation tissue present (Figure 8). The seropurulent drainage deep in the intramuscular structures was no longer present. Since there was adequate removal of nonviable tissue and the quality of the tissue had significantly improved, the WOCN made the decision to transition both wounds to NPWTi-d instilling normal saline solution with a dwell time of 10 minutes and NPWT every two hours at -125 mmHg.

**Figure 9:**
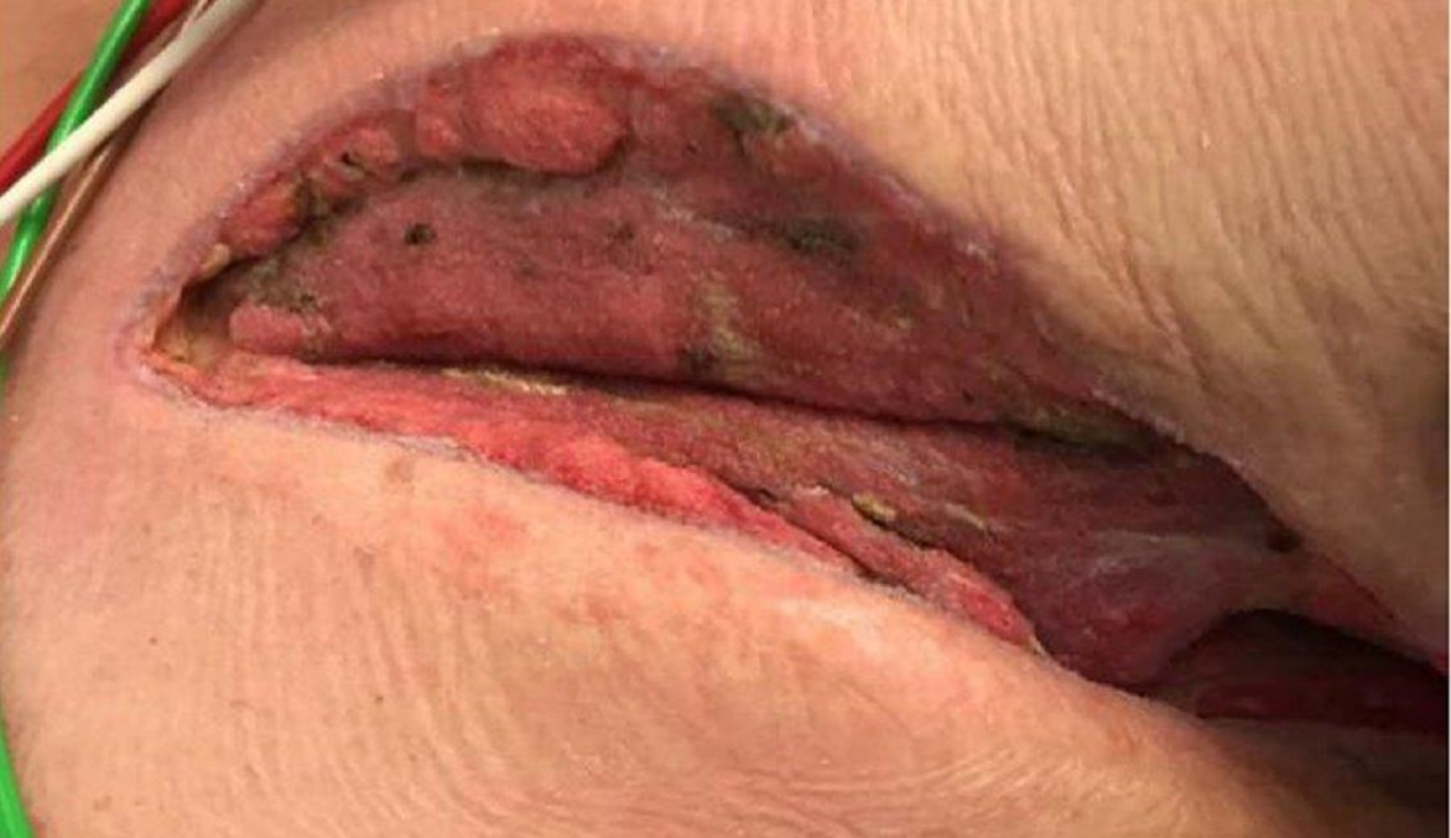
Standard NPWT initiated to left axilla wound

**Figure 10:**
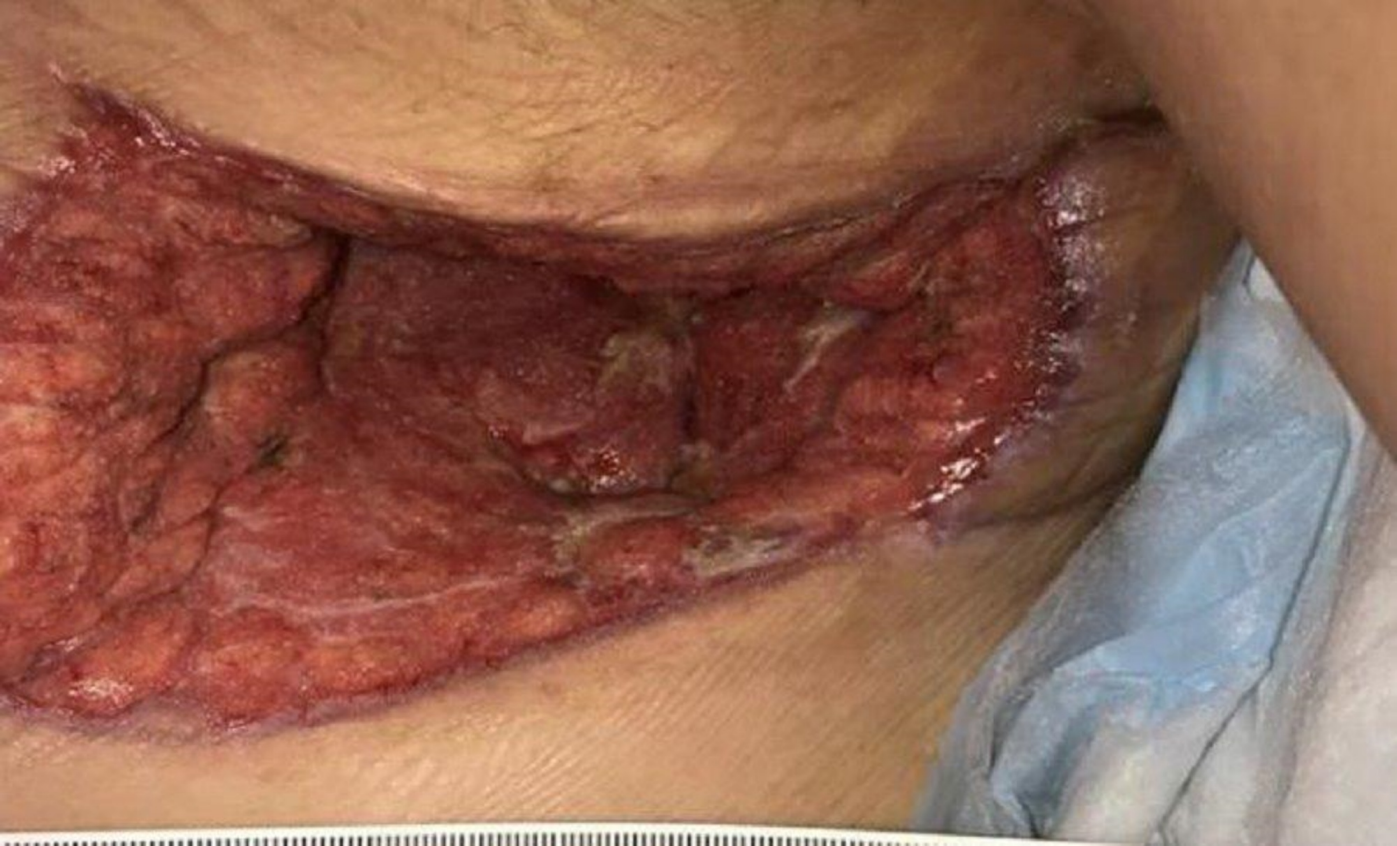
Standard NPWT initiated to left chest wound

For the next two weeks the treatment regimen utilizing NPWTi-d to the wounds continued. The dressings were changed three times a week at the bedside by the WOCN. During initial assessment, the axilla wound measured in total volume 650 cm³ and the chest wound measured 1,216 cm³. After 17 days utilizing the combination of NPWTi-d with and without ROCF-CC, the axilla wound measured in total volume 342.25 cm³ and the chest wound measured 554.4 cm³. This presents a significant decrease in overall measurements. At this time, NPWTi-d was discontinued and standard NPWT with black granufoam was utilized to both wounds at -125 mmHg (Figures 9-10).

**Figure 11:**
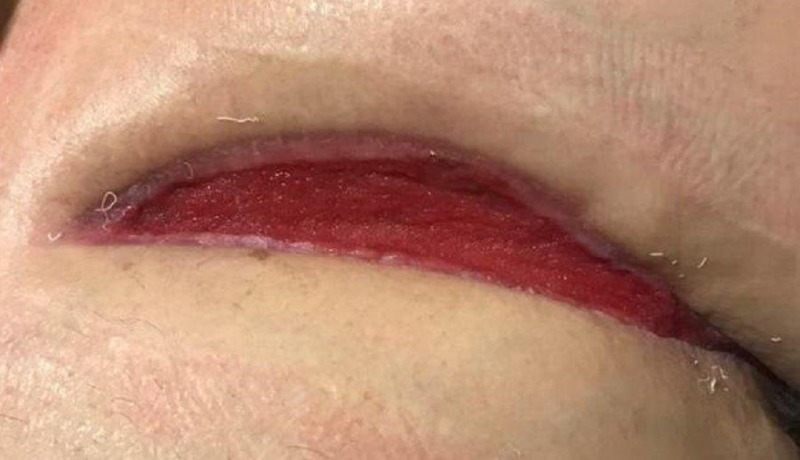
NPWT was discontinued and an antimicrobial alginate dressing was utilized in the left axillary wound

**Figure 12:**
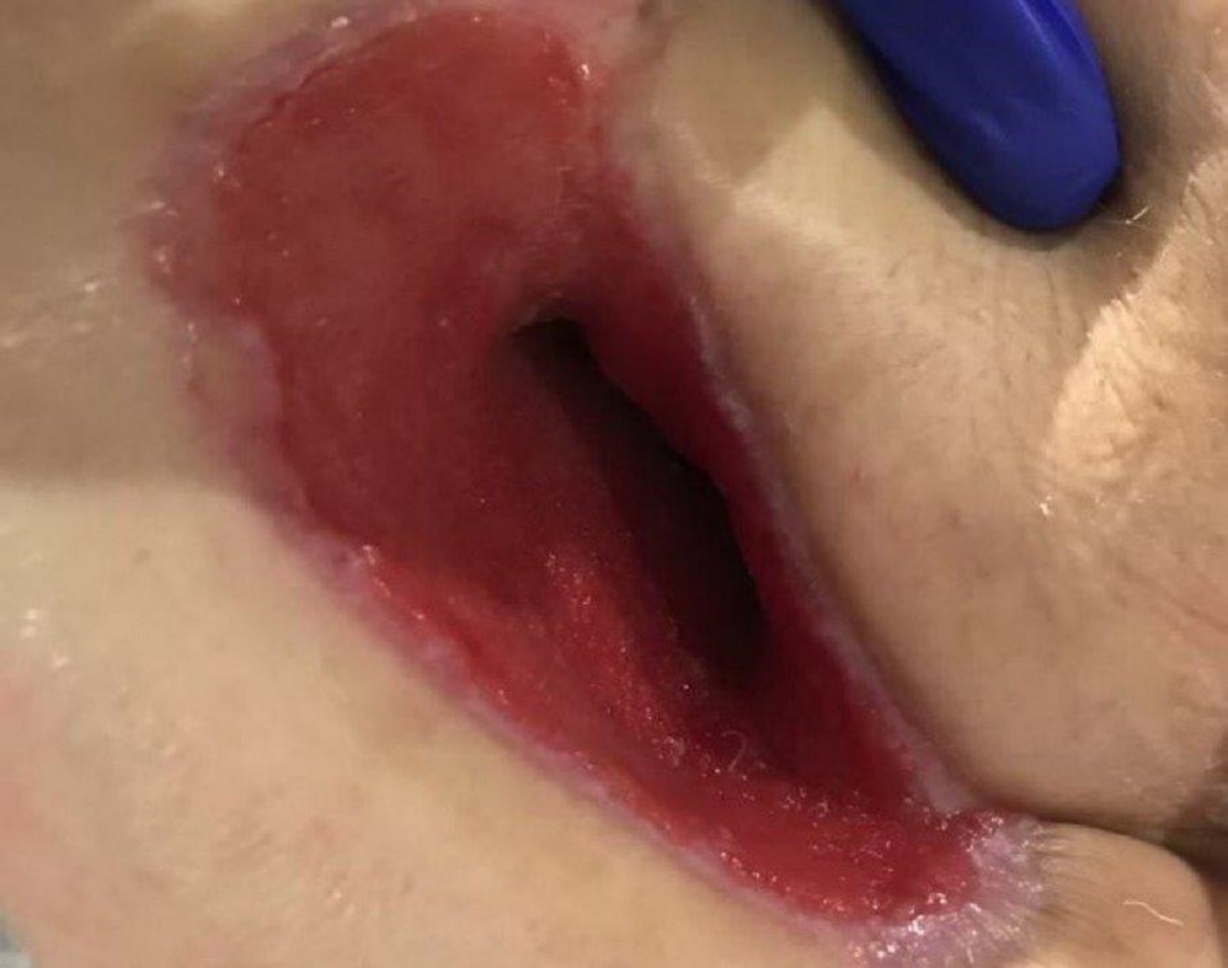
NPWT discontinued and an antimicrobial alginate dressing was utilized for the left chest wound

NPWT was utilized for approximately a month to promote granulation tissue and assist with wound contraction (Figures 11-12). Once the wound had made vast improvements, NPWT was discontinued and a non-adherent antimicrobial alginate dressing (Silvercel™ Non-adherent, Acelity, San Antonio, TX) was lightly packed into the wound bed and covered with a dry sterile dressing. This dressing was then changed three times a week by a visiting nurse association. The wound measurements prior to discharge showed significant improvement in comparison to the wound measurement at presentation, with the left axilla wound measuring 1.5 x 10.5 x 0.3 cm and the left chest wound measuring 3.5 x 9 x 5 cm.

## Discussion

In this case presentation many wound care therapies along with repeat trips to the OR were utilized prior to the use of NPWTi-d. These therapies provided little to no visible improvements in the quality of the wound tissue and also proved to be labor intensive. NPWTi-d with ROCF-CC was utilized initially to solubilize and remove nonviable tissue and infectious material from the wound base. Not only was this goal achieved but the patient did not have to undergo repeat trips to the OR. Once there was a notable decrease in the amount of nonviable tissue in the wound base, the treatment plan was transitioned to NPWTi-d. NPWTi-d was implemented to increase the promotion of granulation tissue and wound contraction. The use of the NPWT family, which ranged from the use of NPWTi-d with ROCF-CC to standard NPWT, proved to decrease the patient’s axilla wound's total volume by 645.28 cm³, which was a 99 percent decrease in dimensions. The same was seen in the dimensions of the chest wound with a total volume decrease of 1,058.54 cm³, which was an 87 percent reduction. The amount of granulation tissue and wound contraction seen in this patient far exceeds the literature's suggestion of 43 percent increase in granulation tissue with the use of NPWTi-d above standard NPWT. Significant improvement in the overall wound assessment was seen in a relatively short period of time considering the extent of the patient’s injuries.

## Conclusions

Necrotizing fasciitis is a quickly developing, life-threatening infection that effects the soft tissue and fascia. Early recognition and appropriate treatment represent the most important factors influencing necrotizing fasciitis patient survival. In critically ill patients with necrotizing fasciitis, NPWT with instillation can minimize toxin absorbance, preserve residual vital tissue, remove nonviable tissue, and effectively close the wound promptly. In this case review, negative pressure wound therapy with instillation has proven to decrease return trips to the OR for debridement, increase healing rates, and decrease length of hospitalization.

The authors recognize that this is a single case study and that the implications for practice cannot be generalized. This does, however, provide an opportunity for further research to see what settings, what patients, and what clinical diagnosis may benefit the most from the advanced wound therapy of NPWTi-d with ROCF-CC.
